# Long-Term Phenological Shifts in Raptor Migration and Climate

**DOI:** 10.1371/journal.pone.0079112

**Published:** 2013-11-01

**Authors:** Mikaël Jaffré, Grégory Beaugrand, Éric Goberville, Frédéric Jiguet, Nils Kjellén, Gerard Troost, Philippe J. Dubois, Alain Leprêtre, Christophe Luczak

**Affiliations:** 1 Centre National de la Recherche Scientifique, Laboratoire d’Océanologie et de Géosciences UMR LOG CNRS 8187, Université Lille 1, Station Marine de Wimereux, Wimereux, France; 2 Muséum National d’Histoire Naturelle, Centre de Recherches sur la Biologie des Populations d’Oiseaux, UMR MNHN-CNRS-UPMC 7204, Paris, France; 3 Department of Biology, University of Lund, Lund, Sweden; 4 Trektellen.org, SOVON Dutch Centre for Field Ornithology, Nijmegen, The Netherlands; 5 Ligue pour la Protection des Oiseaux, Rochefort, France; 6 Université Lille 1, Écologie Numérique et Écotoxicologie, UPRES EA 4515, Villeneuve d’Ascq, France; 7 Université d’Artois, IUFM, Gravelines, France; University of Western Ontario, Canada

## Abstract

Climate change is having a discernible effect on many biological and ecological processes. Among observed changes, modifications in bird phenology have been widely documented. However, most studies have interpreted phenological shifts as gradual biological adjustments in response to the alteration of the thermal regime. Here we analysed a long-term dataset (1980-2010) of short-distance migratory raptors in five European regions. We revealed that the responses of these birds to climate-induced changes in autumn temperatures are abrupt and synchronous at a continental scale. We found that when the temperatures increased, birds delayed their mean passage date of autumn migration. Such delay, in addition to an earlier spring migration, suggests that a significant warming may induce an extension of the breeding-area residence time of migratory raptors, which may eventually lead to residency.

## Introduction

**Figure 1:**
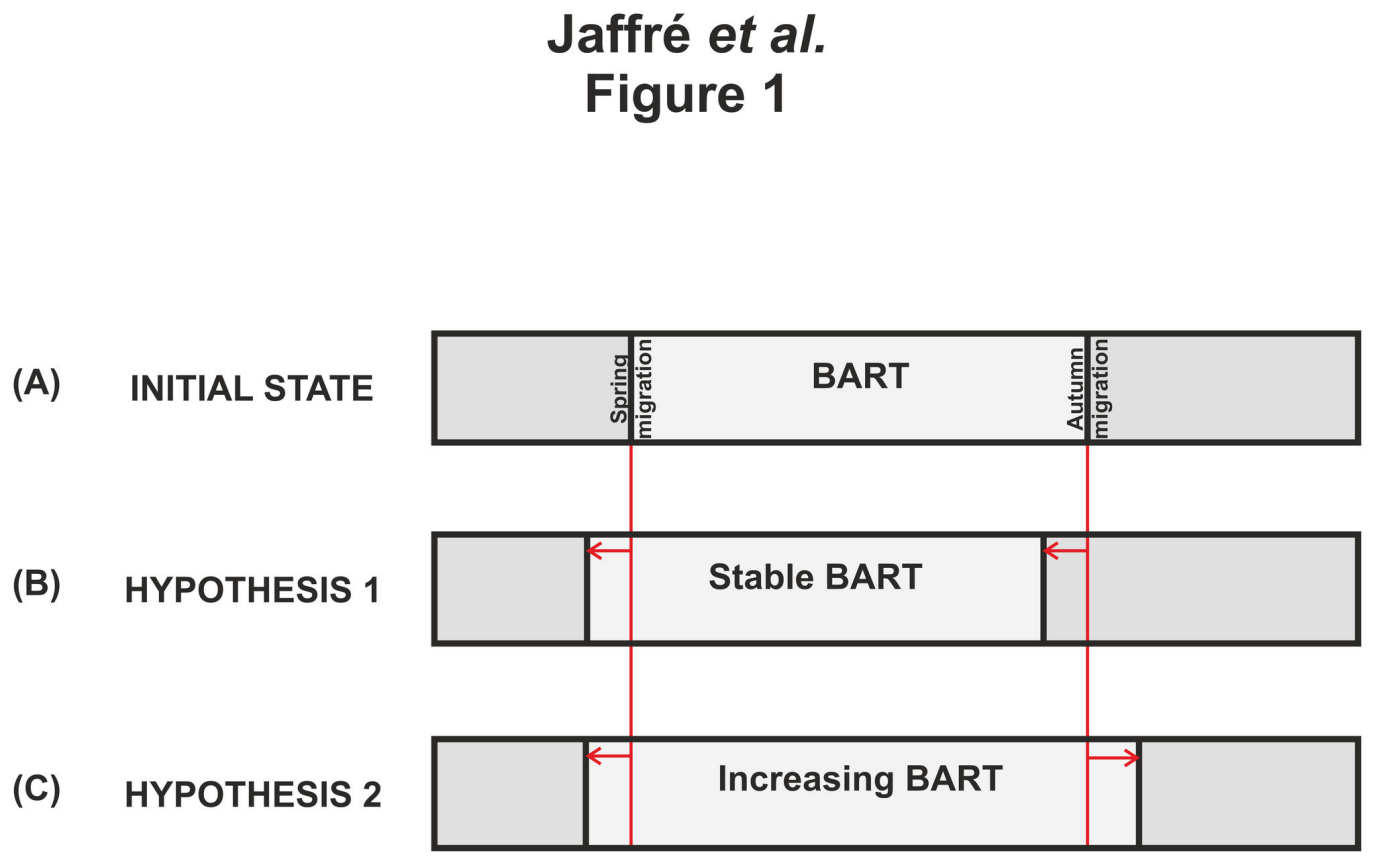
Schematic diagram of the length of the Breeding Area Residence Time (BART) depending on the initial condition and both hypotheses for changes in migration dates. **A**, Initial state: migration dates are unchanged; the length of the BART is stable. **B**, Hypothesis 1: both spring and autumn migration dates occur earlier; the length of the BART is stable but lagged. **C**, Hypothesis 2: spring migration date is earlier, autumn migration date is later; the length of the BART is extended.

Current global climate change has already caused consistent patterns of change in the phenology and biogeography of numerous species, ranging from plants to vertebrates [1,2]. Such changes include pronounced shifts in the timing of the annual cycle events of birds, in particular breeding and migration [3-6]. Most studies have focused on spring migration, showing that migration occurs earlier [4] when temperatures increases [7]. The autumn migration has been less investigated and the responses in the timing of birds seem to be more complex, depending on the ecology and life history traits of the species [8,9]. The fluctuations in the timing of bird migration in response to climate change differs however between long-distance and short-distance migrants [3]. The later birds, spending the winter close to their breeding area, can alter the timing of their migration in response to climate change more rapidly than long-distance migrants [3], which overwinter in the tropics and whose migration is under endogenous control [10]. Several hypotheses have been proposed to explain the variability in post-breeding migration. A first hypothesis assumes an unchanged Breeding-Area Residence Time (so-called BART, Figure 1A) [11] and stipulates that the timing of post-breeding migration takes place earlier [9,12] as a consequence of a premature spring arrival (Figure 1B). A second hypothesis propounds that a more climatically suitable autumn may lead to a later departure because adverse environmental conditions are delayed (Figure 1C) [13,14].

## Materials and Methods

### Biological data

**Figure 2:**
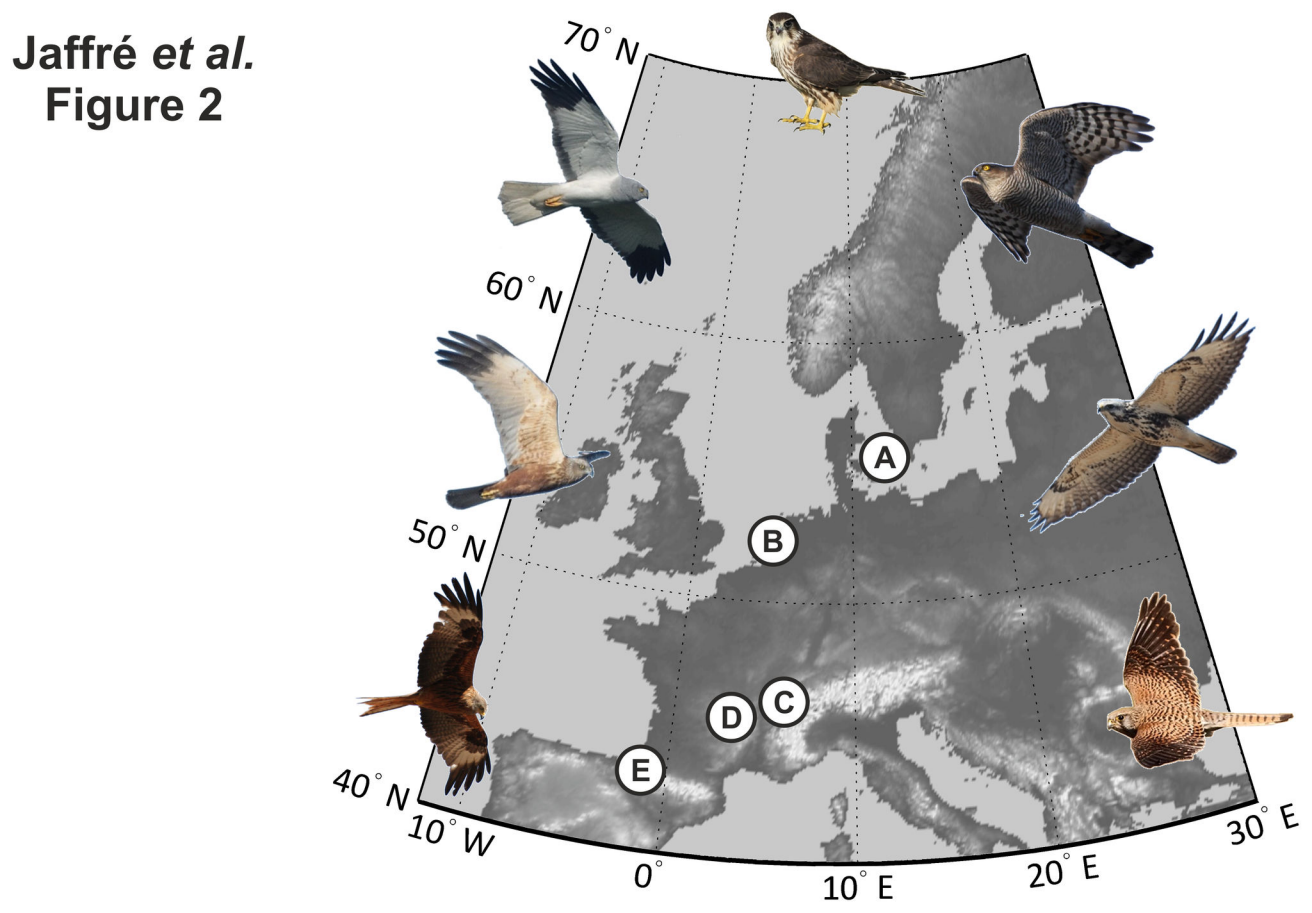
Location of the five sub-regions used in the study: **A**, Sweden. **B**, Netherlands. **C**, French Alps. **D**, Massif Central. **E**, Pyrenees. Illustrations of the seven species of raptor from left to right: red kite, marsh Harrier, hen harrier, merlin, sparrowhawk, common buzzard, and kestrel.

To investigate long-term (1980-2010) changes in raptor migrations in Western Europe, we used data collected on a daily basis at thirteen watchsites located along the most important western european migration flyways. To reduce the number of missing data and optimise seasonal coverage [15], the thirteen watchsites were rearranged into five sub-regions (Figure 2). The bird abundances for each monitoring day and for each sub-region were calculated using the mean of the abundances of the corresponding day in the corresponding sites (Table 1).

**Table 1:** Main characteristics of the sub-regions and watch-sites used in this study.

Sub-region	Count season	Mean days season^-1^ (SD)	Number of years	Names of watch-sites	Latitude	Longitude
Sweden	01Aug.-10Nov.	110 (4)	29	Falsterbo	55.38°N	12.82°E
Netherlands	01Jul.-31Dec.	137 (11)	31	Camperduin	52.73°N	4.64°E
				Kennemerduinen	52.42°N	4.55°E
				Scheveningen	52.12°N	4.28°E
French Alps	15Jul.-30Nov.	96 (36)	26	Les Conches	46.18°N	5.32°E
				Défilé de l’Écluse	46.12°N	5.90°E
Massif Central	01Aug.-31Oct.	92 (26)	24	Saint-Gervais-d’Auvergne	46.03°N	2.82°E
				Montagnes de la Serre	45.66°N	3.09°E
				Col de Baracuchet	45.58°N	3.92°E
				Creste	45.55°N	3.04°E
Pyrenees	15Jul.-15Nov.	113 (8)	30	Col de Lizarrieta	43.26°N	1.62°W
				Redoute de Lindux	43.03°N	1.36°W
				Col d’Organbidexka	43.03°N	1.02°W

We chose seven partial migrant raptor species [16] with a sufficient abundance on the monitored watchsites: eurasian sparrowhawk *Accipiter nisus*, common buzzard *Buteo buteo* (subspecies *buteo*), marsh harrier *Circus aeruginosus* (subspecies *aeruginosus*), hen harrier *Circus cyaneus*, merlin *Falco columbarius*, common kestrel *Falco tinnunculus* and red kite *Milvus milvus*. Only years with at least 75% of days with counts during the migration window (2.5% to 97.5% of the passage) were retained.

### Air temperatures data

Mean monthly air surface temperatures data (1980-2010) originated from the National Oceanic & Atmospheric Administration (NOAA, http://www.esrl.noaa.gov/psd/). These data were retrieved on a grid 2.5°longitude x 2.5°latitude for an area corresponding to Western Europe (12°W - 30°E and 40°N - 70°N).

### Estimation of long-term phenological changes (1980-2010)

The study of long-term phenological changes in raptor post-breeding migration was undertaken using the Mean Passage Date (MPD) [17]. This technique provides accurate estimations of phenological shifts, being robust to variation in sample sizes and imperfect detectability [17].

**Figure 3:**
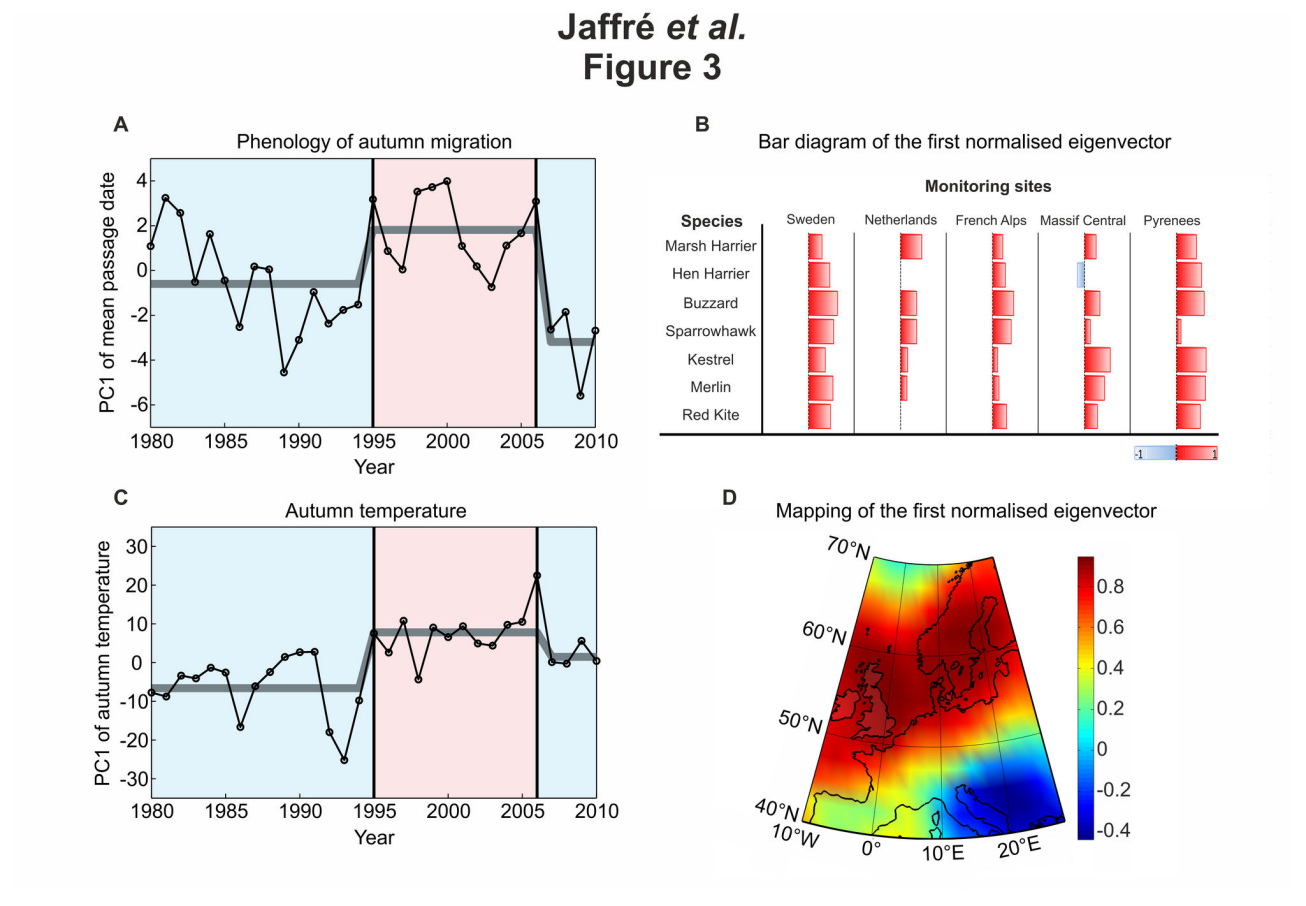
Long-term changes in the timing of autumn migration in relation to autumn temperatures in Europe from 1980 to 2010. **A**, **B**, PCA of the year-to-year changes in the mean passage date of seven species of raptors from 1980 to 2010. (**A**) Long-term changes in the first principal component (PC1), which represents 20.24% of the total variability. Abrupt shifts, identified by the Rodionov’s test, are superimposed (grey line). (**B**) Bar diagram of the first eigenvector normalised between -1 and 1. Negative values are in blue and positive values are in red. **C**, **D**, PCA of the year-to-year changes in the August-October temperatures from 1980 to 2010. (**C**) Long-term changes in the first principal component (PC1), which represents 44.25% of the total variability. Abrupt shifts, identified by the Rodionov’s test, are superimposed (grey line). (**D**) Mapping of the first normalised eigenvector, which shows the correlation between autumn temperatures and the first component. Negative values are in blue and positive values are in red.

Only a few statistical techniques exist to analyse such complex tables, e.g. co-inertia analysis [18] and three-mode principal component analysis (PCA; [19]). The later method was applied here. The three-mode PCA first calculates 3 classical PCAs on 2-dimensional tables after having transformed one table to ensure that the total inertia is identical in each mode [20]. The analysis then relates the different modes by assessing a core matrix calculated from the eigenvectors of each mode (see Beaugrand et al. 2000 [20] for a detailed description of the algorithm). In the present study, the application of this statistical technique allowed in a single analysis (1) the characterisation of temporal changes in the timing of autumn migration by the examination of the first two principal components (PCs; Figure 3A and Figure S1A) and (2) both the identification of species that contribute to the changes and the recognition of sites mainly influenced by the temporal patterns (associated normalised eigenvectors – Figure 3B and Figure S1B). To assess long-term spatial and temporal changes in the MPD of the seven species in western Europe, we only calculated one standardised PCA (i.e. subtracting the mean and dividing by the standard deviation (SD) to give all parameters the same variation) on the deployed 3-way matrix 31 years x (7 species x 5 sub-regions), which represents the first stage performed in a three-mode PCA [20-22].

### Long-term changes in air temperatures

To evaluate the influence of climate on the phenology of post-breeding migration, we examined long-term changes in autumn and spring temperatures in Western Europe by standardized PCA (Joliffe 1986). The analyses were performed in the spatial domain ranging from 10°W to 30°E and from 40°N to 70°N and data were averaged on a grid of 2.5° longitude x 2.5° latitude for each year of the period 1980-2010. We selected (1) August to October temperatures (autumn) because this period corresponds to the time between the emancipation of young and the migration period and (2) April temperatures (spring) as the timing of breeding during this month could be linked with the timing of autumn migration [23]. We then performed two standardised PCAs with the double objective of identifying major long-term changes in both autumn and spring temperatures (examination of the first PCs, Figure 3C) and locating their geographical pattern (mapping of the first normalised eigenvectors, Figure 3D).

### Identification and quantification of temporal discontinuities

**Figure 4:**
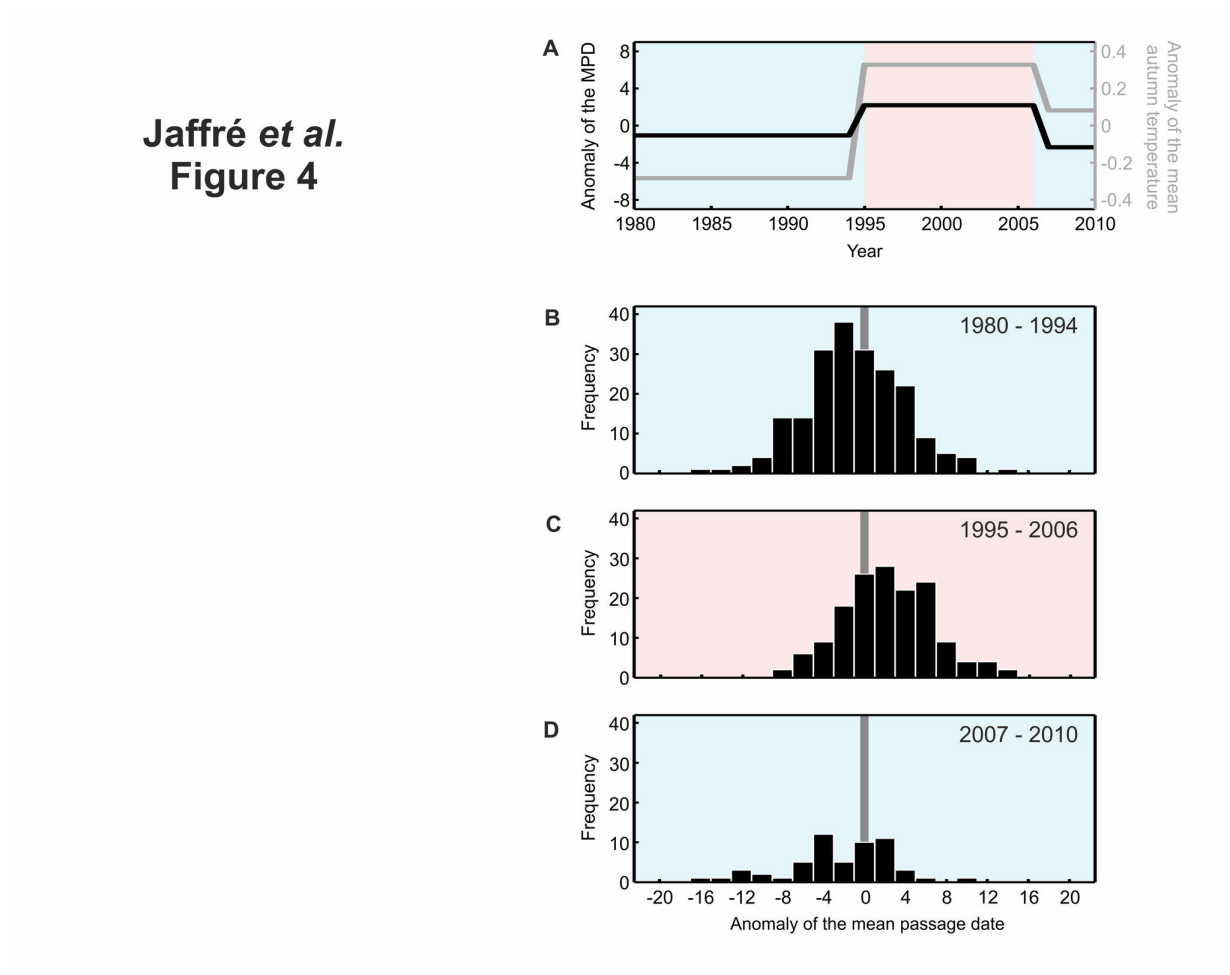
Effect of the abrupt shifts in autumn temperatures on the timing of migration. **A**, mean anomalies of autumn temperatures and Mean Passage Date (MPD; in day) for all periods revealed by the Rodionov’s test (see Figure 2). Both autumn temperatures and MPD are expressed in anomalies with respect to the period 1980-2010. b, c, d, Repartition of the anomalies of MPD during (**B**) the first period 1980-1994 (T1=12.29°C), (**C**) the second period 1995-2006 (T2=12.91°C) and (**D**) the third period 2007-2010 (T3=12.66°C).

We applied the Rodionov's sequential algorithm to detect the temporal discontinuities (1980-2010) occurring in the first PCs originating from the PCA performed on post-breeding migration (Figure 3A and Figure S1A), autumn temperatures (Figure 3C) and spring temperatures (Figure S1C). We used a first-order autoregressive model to consider temporal autocorrelation [24], a probability threshold of p=0.1 and cut-off length of l=10 to control the magnitude and the scale of the shift, respectively. To consider outliers above 1 standard deviation, the Huber's weight parameter was fixed to h=1. As guidance for choosing parameters p and l, setting parameters were adjusted following the recommendations of previous studies to detect shifts with meaningful environmental and biological implications [24-26]. The choice of a significance level p=0.1 is a good compromise between robustness of the detected shift and the flexibility of the detection [24,25]. A cut-off length of l=10 discounts regimes of shorter length to detect only statistically significant long regime shifts depending on the studied period (i.e. 30 years - [26]). A Huber parameter of h=1 is such that the number of detected shifts remains stable for higher values of h. More details about the methods and parameters it takes can be found at www.beringclimate.noaa.gov/regimes/. The magnitude of the shifts was subsequently quantified by selecting Sweden and Pyrenees (Table 1), sub-regions for which complete datasets exist for all considered raptors. For each regime, MPD anomalies were assessed for every year and species with respect to the period 1980-2010. A histogram was then calculated to reveal the statistical distribution of all anomalies for each regime (Figure 4). The average anomalies of autumn temperatures and post-breeding migration were also assessed for each regime (Figure 4A).

### Relationships between long-term changes in post-breeding migration and temperatures

Linear correlations were calculated to assess the relationships between long-term changes in the first two PCs of post-breeding migration and changes in both April and autumn temperatures. The probabilities of the correlations were corrected to account for temporal autocorrelation [21].

## Results and Discussion

Year-to-year changes in the first Principal Component performed on MPD (PC1; 20.24% of the total variability) revealed a period of relative early passage until 1994, followed by a period of later bird migration until 2006 and finally a return to an earlier MPD (Figure 3A). The examination of the first normalised eigenvector indicates that the long-term phenological shifts exhibited by PC1 concerned virtually all species and sub-regions (Figure 3B). The Rodionov's test applied on PC1 shows that short-distance migrant raptors have abruptly delayed their MPD in Western Europe in 1995 and returned to an earlier autumn migration in 2006. The second Principal Component (PC2; 13.03% of the total variability) revealed a tendency for these birds (except red kite *Milvus milvus*) to an earlier MPD interrupted in 2006 (Figure S1A). The examination of the second normalised eigenvector indicates an opposition between the long-term phenological shifts in northern sites (the Netherlands, the French Alps and to a lesser extent in Sweden) on one side, and in southern sites (the Massif Central and Pyrenees) on the other side (Figure S1B).

Mapping of the first eigenvectors of the PCA performed on temperatures in Western Europe indicated that the trends detected by the PCAs reflect well changes in autumn temperatures in the north-western part of Europe (Figure 3D) and mostly changes in spring temperatures in the northern part of Europe (Figure S1D). 

We subsequently applied the Rodionov's test on the PC1 of autumn temperatures (44.25% of the total variability) and on the PC1 of spring temperatures (34.32% of the total variability) to detect temporal discontinuities. For autumn temperatures, the analysis detected the same temporal discontinuities than those identified previously by the first PC of the MPD (see Figure 3, A and C): a relative cold regime until 1994, followed by a warmer regime until 2006 and a return to cooler conditions from 2007 to 2010 (Figure 3C). For spring temperatures, the analysis only found one single temporal discontinuity: in 1999. A relative cold regime occurred until 1998 and was subsequently followed by a warmer regime (Figure S1C). No synchronous discontinuities were detected between the second PC of the MPD and the first PC of spring and autumn temperatures. We also tested for linear relationships between both spring and autumn temperatures and the timing of post-breeding migration. For PC1 of the MPD, no linear correlation was detected between either spring (r=-0.23, PACF=0.28) or autumn (r=0.31, PACF=0.18) temperatures. For PC2 of the MPD, while no linear correlation was detected with autumn temperatures (r=0.44, PACF=0.12), a significant relationship was found with spring temperatures (r=-0.55, PACF=0.03). These results provide evidence that, although part of phenological changes were correlated with the spring temperatures (i.e. linear relationship with the CP2 of the MPD), most short-distance migrants responded abruptly to pronounced shifts in autumn temperatures during the period 1980-2010 (i.e. synchronous discontinuities with the CP1 of the MPD; Figure 3A and Figure 3C), suggesting that they adjusted their postnuptial migration timing to the thermal regime.

Three periods were detected: 1980-1994, 1995-2006 and 2007-2010 (Figure 4A). We quantified the magnitude of the shifts (1994-1995 and 2006-2007) by calculating the average of MPD and autumn air temperature for each period (Methods). While between 1980-1994 and 1995-2006 the autumn temperatures increased by 0.61°C, post-breeding migration expressed as MPD was delayed for all species but one (marsh harrier) from 0.2 to 8.9 days (mean = 3.23 days, or 5.28 days/°C; Figure 4). Marsh harrier exhibited a negative response probably because northern populations of this species are long-distance migrants [27]. This result is in agreement with a study, which showed that post-breeding migration became earlier during the last three decades for this species [12]. Between 1995-2006 and 2007-2010, the autumn temperatures diminished by 0.25°C and paralleled an earlier MPD ranging from 1.6 to 8.9 days (mean = -4.52 days, or -18.28 days/°C; Figure 4). Sparks and Menzel reported that a 1°C increase in temperature could advance arrival time by days [28]. Therefore, our observed changes of 5.28 days/°C (first shift) and -18.28 days/°C (second shift) are less conservative and suggest a high sensitivity of post-breeding migration of short-distance migrant raptors to climate-induced changes in autumn temperatures over their breeding area. It has been shown that genetic factors play an important role in the control of migratory behaviour of birds [29] and that this behaviour is partly hereditary [30]. Such a result implies that rapid changes in migratory behaviour can occur, especially for species with short generation time. However, the birds studied here are long-lived species and have a long generation time (e.g. five years for common buzzard *Buteo buteo*, i.e. up to six generations during the three decades [23]), which is inconsistent with the hypothesis of a positive selection of late migrants. Hence, abrupt shifts observed in this study are probably not a micro-evolutionary response of species. This statement is in accordance with the weak evolutionary potential found in raptors [31]. Instead, the rapid phenological response of short-distance migrant raptors observed here in autumn is probably a phenotypic response of these birds to improved foraging and environmental conditions at middle-high latitudes.

Other studies found a relationship between earlier autumn migration and spring temperatures [8,9,14]. Here we also found a significant correlation between the second PC, which exhibit an earlier MPD, and the increase of spring temperatures. This earlier passage of raptors is mostly prominent over northern sub-regions, where birds are known to be more strictly migratory. This means that the first hypothesis (unchanged BART) is also valid with our data, but is of lesser importance compared to the second hypothesis of increased BART (13.03 < 20.24% of the total variability). Most studies, including ours, failed to find a link between the autumn MPD of birds and the autumn temperatures on the breeding grounds [14,32]. However, our analysis on temporal discontinuity revealed an abrupt response of birds to changes in autumn temperatures. Our results suggest that not only BART may increase as a result of earlier spring arrival due to warmer conditions in spring [23,33] but also because European short-distance migratory raptors tend to delay their departure as a result of warmer conditions in autumn. In this context, it is likely that global warming will enable several species to reside on their breeding grounds some favourable years. According to the Intergovernmental Panel on Climate Change, a moderate scenario (A1B scenario) of global warming will increase the temperatures between 2.2°C and 5.3°C in Europe by 2100 [34]. Assuming a linear relationship between BART and autumn temperatures, it is plausible that such a rise in temperatures leads to a mean extension ranging from 11.6 (+2.2°C) to 28.0 (+5.3°C) days of BART in autumn for the seven studied species and by [32.1 - 77.4] days for the extreme case of hen harrier. Improved foraging in winter could enable birds overwintering closer to their breeding range which therefore reduce their migration distance [35,36]. The present manuscript adds a phenological aspect to the distance reduction, and both phenological changes and reduction of the migration distance suggest that climate change, associated with a decrease in seasonality, could have potential implications for some birds to forgo migration altogether, leading to residency [37]. This hypothesis is supported by the ‘Shifting Home’ model [36], a model of the origin of the migration system which suggests that the periods of global cooling during the Cenozoic drove shifts of the wintering area of some Palearctic birds and led to the emergence of migration [36]. In an opposite climatic context (a global warming instead of a global cooling), we observe an opposite feature. While the ‘Shifting Home’ model proposes that decreasing winter temperatures led birds to migrate towards more favourable areas, the current increase in temperature could result in a reduction of the migratory behaviour, resulting in the residency of species.

## Supporting Information

Figures S1 **Long-term changes in the timing of Autumn migration in relation to Spring temperatures in Europe from 1980 to 2010.** A, B, PCA of the year-to-year changes in the mean passage date of seven species of raptors from 1980 to 2010. (A) Long-term changes in the second principal component (PC2), which represents 13.03% of the total variability. Abrupt shifts, identified by the Rodionov’s test, are superimposed (grey line). (B) Bar diagram of the second eigenvector normalised between -1 and 1. Negative values are in blue and positive values are in red. C, D, PCA of the year-to-year changes in the April temperatures from 1980 to 2010. (C) Long-term changes in the first principal component (PC1), which represents 34.32% of the total variability. Abrupt shifts, identified by the Rodionov’s test, are superimposed (grey line). (D) Mapping of the first normalised eigenvector, which shows the correlation between spring temperatures and the first component. Negative values are in blue and positive values are in red.
(TIF)
