# Supplementary figures and images for: Long-Term Phenological Shifts in Raptor Migration and Climate
Source: PLoS One. 2013 Nov 1;8(11):e79112. doi: 10.1371/journal.pone.0079112 (PMC3815123; doi:10.1371/journal.pone.0079112)

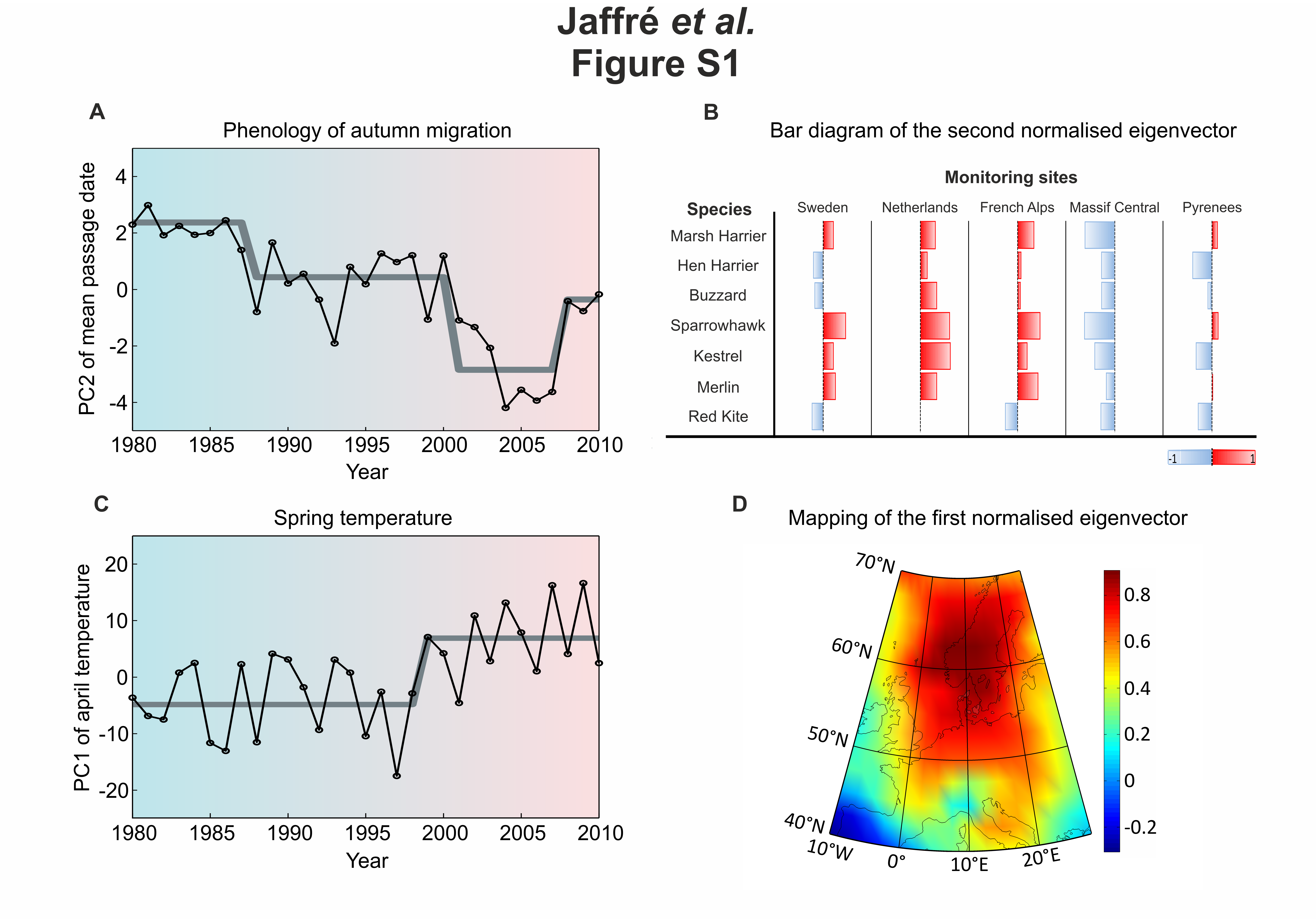

Supplement: Figures S1 — Long-term changes in the timing of Autumn migration in relation to Spring temperatures in Europe from 1980 to 2010. A, B, PCA of the year-to-year changes in the mean passage date of seven species of raptors from 1980 to 2010. (A) Long-term changes in the second principal component (PC2), which represents 13.03% of the total variability. Abrupt shifts, identified by the Rodionov’s test, are superimposed (grey line). (B) Bar diagram of the second eigenvector normalised between -1 and 1. Negative values are in blue and positive values are in red. C, D, PCA of the year-to-year changes in the April temperatures from 1980 to 2010. (C) Long-term changes in the first principal component (PC1), which represents 34.32% of the total variability. Abrupt shifts, identified by the Rodionov’s test, are superimposed (grey line). (D) Mapping of the first normalised eigenvector, which shows the correlation between spring temperatures and the first component. Negative values are in blue and positive values are in red. (TIF) [file pone.0079112.s001.tif]
